# Complement System–Related Genes in Diabetic Nephropathy: Screening for Potential Targets of the Mechanism

**DOI:** 10.1155/jdr/7117751

**Published:** 2026-07-05

**Authors:** Lifang Wei, Ye Li, Yuhui Lu, Minmin Xu, Yue Yu, Lijie Li, Min Lin, Lifei Fan, Jing Cai

**Affiliations:** ^1^ Department of Nephrology, The Third People′s Hospital Affiliated to Fujian University of Traditional Chinese Medicine, Fuzhou, Fujian, China; ^2^ Fujian University of Traditional Chinese Medicine, Fuzhou, Fujian, China, fjtcm.edu.cn; ^3^ College of Traditional Chinese Medicine, Fujian University of Traditional Chinese Medicine, Fuzhou, Fujian, China, fjtcm.edu.cn; ^4^ The Second People’s Hospital Affiliated to Fujian University of Traditional Chinese Medicine, Fuzhou, Fujian, 350003, China, fjtcm.edu.cn; ^5^ Clinical Research Center for Traditional Chinese Medicine on Glucolipid Metabolic Disorders of Fujian Province, Fuzhou, Fujian, China

**Keywords:** complement system, diabetic nephropathy, diagnosis, Mendelian randomization, transcriptomics

## Abstract

Diabetic nephropathy (DN) is the most common complication of diabetes, with immune‐mediated inflammation playing a significant role in its pathophysiology. The complement system, a key proinflammatory factor, is implicated in DN. This study was aimed at identifying diagnostic biomarkers related to the complement system in DN using bioinformatics methods. We analyzed three datasets (GSE96804, GSE104948, and GSE1009) from public databases, employing differential expression analysis and machine learning to identify complement system–related genes (CSRGs) as potential biomarkers. A diagnostic nomogram was constructed based on these genes, and immune microenvironment differences between the DN and control groups were explored. Gene interaction networks, enrichment analysis, and drug predictions were also conducted. Mendelian randomization (MR) was used to examine the causal links between identified biomarkers and DN. Our analysis identified five biomarkers (CKB, ANXA1, HSPA1L, CYP27B1, and XYLT1) associated with DN. A diagnostic nomogram based on these biomarkers showed high accuracy (model correction slope close to 1 and AUC close to 1). Immune infiltration analysis revealed significant differences in immune cell subsets between the DN and control groups. Gene set variation analysis (GSVA) indicated that the oxidative phosphorylation (OXPHOS) pathway was activated in CKB, HSPA1L, CYP27B1, and XYLT1 while inhibited in ANXA1. MR confirmed HSPA1L as a risk factor for DN (OR = 1.625, 95% CI: 1.272–2.076, *p* = 9.96e − 05). In conclusion, these five CSRGs may play significant roles in DN progression, providing a foundation for further research into DN pathogenesis and potential molecular markers for clinical diagnosis.

## 1. Introduction

Diabetic nephropathy (DN), recognized as the predominant cause of chronic kidney disease (CKD) and end‐stage kidney disease (ESKD), is responsible for approximately 50% of cases globally and has emerged as an escalating concern in global health [1–3]. DN is a significant complication of diabetes characterized by kidney damage. With the rapid increase in the prevalence of diabetes, the incidence of DN is also increasing, with approximately 20%–50% of individuals with Type 2 diabetes (T2D) suffering from DN [4]. Current studies suggest that the chronic hyperglycemic environment in diabetes leads to excessive production of proinflammatory mediators, including the activation of complement proteins. Emerging evidence indicates that increased activation of the complement system in diabetic animal models and clinical samples may serve as a biomarker for the progression of DN [5–12]. Despite some progress in treatment—such as blood sugar control, blood pressure management, lipid regulation, and renal protection strategies—current methods are still unable to prevent the progression of DN [13, 14].

Complement component C3 is activated through three different mechanisms that activate the complement system: the lectin pathway, the classical pathway, and the alternative pathway. It is essential for defending against pathogens and maintaining homeostasis in the body [15]. In DN, the activation of the complement system is often triggered by hyperglycemia, leading to the formation of advanced glycation end‐products that can directly activate complement components like C1q [12, 16]. Elevated levels of complement components in DN patients correlate with renal damage and worsening kidney function [8, 17]. Additionally, urinary complement activation products, including C3a, C5a, and C5b‐9, have been shown to increase significantly during the proteinuria stage of DN and correlate with the severity of renal tubular damage. These findings suggest that urinary complement activation may contribute to renal tubular interstitial damage, particularly in the later stages of DN [18]. Understanding these mechanisms not only enhances our knowledge of DN but also opens avenues for potential therapeutic strategies aimed at modulating complement activity to mitigate kidney damage in diabetic patients.

In this study, bioinformatics approaches were used to identify diagnostic and therapeutic biomarkers related to the complement system in DN. The findings are aimed at providing a theoretical basis for the clinical diagnosis and treatment of DN by uncovering the potential mechanisms underlying complement activation and its role in disease progression.

## 2. Materials and Methods

### 2.1. Data Source

The transcriptome data of DN were obtained from the GEO database. GSE96804, which encompasses glomerular tissue from 41 DN patients and 20 control samples, was utilized as the training set (platform GPL17586). Simultaneously, samples from two platforms within the GSE104948 dataset—specifically, GPL22945 (18 control samples and seven DN samples) and GPL24120 (three control samples and five DN samples)—were merged with the GSE1009 dataset (platform GPL8300: three control samples and three DN samples) to form the validation set [19]. Batch effects across these merged datasets (comprising 15 DN samples and 24 control samples) were eliminated using the ComBat function implemented in the “sva” R package (v3.46.0). A total of 12,804 genes annotated as complement system–related were retrieved from the GeneCards database (v5.20). Given that GeneCards integrates evidence from multiple sources and may include genes with both direct and indirect associations, we applied a relevance score threshold of > 7 to retain genes with relatively stronger associations, yielding 2469 candidate complement system–related genes (CSRGs) [20]. The detailed characteristics and analytical roles of all datasets included in this study are summarized in Supporting Information 1: Table S1. For the microarray data, background correction, quantile normalization, and probe summarization were performed before downstream analysis. Probe‐to‐gene mapping was conducted according to the annotation files provided by the corresponding platforms. When multiple probes were mapped to the same gene symbol, the average expression value was used.

### 2.2. Differential Expression Analysis

DN and control samples in the GSE96804 dataset were analyzed using the R software limma package (v3.60.4) [21] to screen for differentially expressed genes (DEGs), with the filtering conditions: |log2*f* *o* *l* *d* *c* *h* *a* *n* *g* *e* (*F* *C*)| > 0.5, *p*.adjust < 0.05. Volcano and heatmaps were plotted using ggplot2 (v3.5.1) [22] and ComplexHeatmap (v2.20.0) [23], respectively, to show the DEGs. A module of expression pattern genes was constructed on GSE96804 using weighted gene coexpression network analysis (WGCNA) [24]. Before network construction, genes were ranked according to median absolute deviation (MAD), and the top 75% most variable genes were retained for WGCNA. The initial step was to ascertain whether outlier samples required removal. Subsequently, the optimal soft‐thresholding power was selected based on the scale‐free topology criterion, and the network type was set to “unsigned.” A topological overlap matrix was then constructed. Subsequently, a systematic clustering tree was constructed for the purpose of classifying the highly coexpressed genes into the same module. The minimum number of genes per module was set to 80, and similar modules were merged using MEDissThres = 0.4, whereas all other parameters were kept at their default settings. The module genes with the highest correlation with DN were identified. Ultimately, the intersection of DEGs, DN‐related module genes, and CSRGs was employed to identify the differentially expressed complement system–related genes (DE‐CSRGs) associated with DN.

### 2.3. Enrichment Analysis of DE‐CSRGs

The enrichment analysis of Gene Ontology (GO) and Kyoto Encyclopedia of Genes and Genomes (KEGG) was conducted by clusterProfiler (v4.12.0) [25] (*p* < 0.05). The results of the analysis were visualized using the R software package ggpubr (v0.6.0) [26], which was employed to display the three GO enrichment categories and the Top 10 entries in KEGG.

### 2.4. Machine Learning

Subsequently, three distinct algorithms, namely, least absolute shrinkage and selection operator (Lasso), support vector machine–recursive feature elimination (SVM‐RFE), and Boruta, were employed for further screening based on the DE‐CSRGs. A Lasso analysis was conducted using the glmnet package (v4.1‐8) [27], with fivefold cross‐validation implemented [28]. The parameters were set as follows: family = “binomial” and type.measure = “default.” Subsequently, we used the e1071 package (v1.7‐14) and the caret package in the R language [29] to obtain the importance and ranking of the features in the DE‐CSRGs using the SVM‐RFE method. For this analysis, fivefold cross‐validation was adopted, while other parameters remained at their default settings. During the process, the error rate and accuracy corresponding to each iterative combination were recorded. The combination with the lowest error rate and accuracy was selected as the final model, and the genes associated with this optimal combination were extracted as potential candidate genes. In addition, Boruta analysis—an approach analogous to the random forest classification algorithm—was conducted to further analyze the DE‐CSRGs using the Boruta package (Version 8.0.0) [30] in R. Here, the dotTrace parameter was set to 2, and all other parameters were used with their default values. The R language package ggvenn (v0.1.10) [31] was utilized to delineate the intersection of genes obtained by the three machine learning algorithms as candidate genes.

### 2.5. Screening of Biomarkers

We used box plots to show differences in expression of candidate genes in disease and control samples and performed the Wilcoxon rank‐sum test between groups. Subsequent to batch effect removal, an empirical Bayes test was applied to the integrated dataset to examine between‐group variation. Genes with the same expression trend and significant differences in the GSE96804 and merged validation set were selected as biomarkers.

### 2.6. Construction of a Nomogram

A nomogram model was constructed by the rms package (v6.8‐1) [32]. The gene expression levels within the model were assigned a score, with the total score being the sum of all the individual gene scores. Subsequently, in order to assess the precision of the nomogram model, calibration curve and receiver operating characteristic (ROC) curve were constructed to align with the nomogram.

### 2.7. Construction of Coexpression Network and Gene Set Enrichment Analysis (GSEA)

To further investigate the interactions among biomarkers, the GeneMANIA database (http://genemania.org/) was employed for the analysis of biomarkers and the construction of a network. Concurrently, GSEA was performed using the clusterProfiler package (v4.12.0) in R. Specifically, in the training set, the correlation between each biomarker and all genes was calculated, and genes were ranked according to the magnitude of the correlation coefficients. Subsequently, GSEA was conducted based on the MSigDB “KEGG” background gene set (c2.all.v2023.2.Hs.symbols.gmt). Pathways with |*N* *E* *S*| > 1 and adjusted *p* value (*p*.adjust) < 0.05 were considered significantly activated or inhibited.

### 2.8. Immune Infiltration, Immune Checkpoint, and Regulatory Network

The ImmuCellAI package (v0.1.0) [33] for the R language was employed to assess the infiltration of 25 distinct immune cell types between DN and control groups in a training cohort. Immune cell types with zero infiltration in more than 50% of samples were excluded from downstream analysis. To compare the results between the two groups, the Wilcoxon test was performed, and the *p* values were adjusted by the false discovery rate (FDR) method. The R software package ComplexHeatmap (v2.20.0) [23] was employed for the purpose of mapping the abundance of immune cells in DN and control samples. A Spearman correlation analysis was conducted between immune cells and biomarkers, with the results visualized using the R package ggcor (v0.9.8.1) [34]. Moreover, Pearson′s correlation analysis was employed to examine the expression of 79 immune checkpoint molecules [35] in the DN sample group, with the resulting correlation graph generated using the R package ggpubr (v0.6.0) [26].

To explore potential regulatory relationships of biomarkers, the TFtarget database was employed to predict the transcription factors (TFs) targeting biomarkers. Furthermore, the miRDB database (v6.0) was utilized to predict microRNAs (miRNAs) that were regulated with biomarkers, and miRNAs supported by CHP‐SEQ data and with an interaction score of more than 80 were screened to construct regulatory networks. Finally, the network diagram was visualized using the Cytoscape software (v3.10.2) [33].

### 2.9. Prediction of Related Diseases and Targeted Drugs Based on Biomarkers

The NetworkAnalyst platform is capable of predicting the onset of diseases and multiple chronic complications associated with specific biomarkers. Concurrently, the DGIdb database (https://dgidb.genome.wustl.edu/) was employed to predict potential drugs targeting biomarkers for DN. And the network was mapped using Cytoscape (v3.10.2) [33] software. The corresponding protein structure of the biomarker was obtained from the PDB (https://www.rcsb.org/) and the AlphaFold Protein Structure Database (https://alphafold.ebi.ac.uk/) databases. Next, the three‐dimensional molecular structure of the corresponding drug compound was obtained from the PubChem database (https://pubchem.ncbi.nlm.nih.gov/) and optimized using the MM2 force field in Chem Bio3D. The optimized structure was saved in PDB format. Additionally, the AutoDock Vina program was employed to dock the protein structure of the biomarker to the corresponding drug compound, and the binding energy between the ligand and the receptor was calculated [36]. The results of the docking process were visualized using PyMOL software (v2.6.0).

### 2.10. Selection of Instrumental Variables (IVs) for Mendelian Randomization (MR) Analysis

In order to investigate the causal relationship between biomarkers and DN, MR analysis was implemented with biomarkers as exposure factors and DN as outcome events. The GWAS data of the DN and eQTL GWAS of biomarkers were determined by the IEU OpenGWAS database (https://gwas.mrcieu.ac.uk/). The finn‐b‐DM_NEPHROPATHY dataset comprised 213,746 samples (3283 cases and 210,163 controls), with a total of 16,380,453 SNPs and an ethnicity designation of European, and was used as the dataset of DN. In order to ensure the validity and reliability of the results of the study, the MR dataset was based on three fundamental assumptions. (1) Association hypothesis: SNPs serve as IVs for exposure. (2) Independence assumption: These IVs are not influenced by confounding factors. (3) Exclusivity assumption: The effect of the SNP on outcome was mediated by the exposure factor alone.

The TwoSampleMR (v0.6.6) [37] package was employed to perform MR analysis for DN and biomarkers, and extract_instruments was employed to read and screen the IVs (SNPs). *p* < 1∗10^−5^ was termed as a screening criterion to identify the IVs that were significantly correlated with the exposure factor. In our current research, we noted that the number of significant SNPs was insufficient, thus failing to meet the basic requirements for MR investigations. Therefore, we utilized a more lenient *p* value threshold of *p* < 1∗10^−5^ to increase the number of instrumental factors and maintain statistical power [38, 39]. Furthermore, biomarkers with a number of IVs less than three were excluded from MR analysis. SNPs with linkage disequilibrium (LD) were removed with parameter settings: *r* ^2^ = 0.001; kb = 100; clump = TRUE. Additionally, weak IVs with an *F*‐statistic value less than 10 were also removed. Finally, SNPs that exhibited a significant correlation with DN were removed using the R package TwoSampleMR function harmonise_data [37], with the objective of unifying the effect and effect size.

### 2.11. Exploring the Causal Association Between Biomarkers and DN

MR analysis was conducted employing the MR function, combining five distinct algorithms: MR‐Egger, weighted median, inverse variance weighted (IVW), simple mode, and weighted mode [40]. The robustness of the IVW method in detecting causal relationships allowed for the identification of significant associations (*p* < 0.05). In the end, the robustness of the MR results was assessed by sensitivity analyses, including Cochran′s *Q* test for instrument heterogeneity, the MR‐Egger intercept test for directional horizontal pleiotropy, and the leave‐one‐out (LOO) analysis [41].

### 2.12. Statistical Analysis

All analyses were conducted using R packages (Version 4.2.1). The *p* value was less than 0.05, indicating a statistically significant result.

## 3. Results

### 3.1. Selection and Enrichment Analysis of DE‐CSRGs

The differential expression analysis of the training set revealed the presence of 1328 DEGs between the DN and control groups. Of these, 611 DEGs were found to be upregulated, whereas 717 were downregulated (Figure 1A,B). Concurrently, the WGCNA was conducted on the training set to obtain the key module genes of the DN. Subsequently, the cluster analysis demonstrated that the clustering of the samples was satisfactory, and no sample elimination was required (Figure 1C). In order to guarantee the nonscale distribution of genes, the optimal soft threshold of 8 was selected (Figure 1D). Furthermore, the minimum number of genes per gene module was set to 80, in accordance with the standard of the hybrid dynamic shear tree algorithm (dynamic tree cutting). This process yielded a total of eight modules (Figure 1E). The module with the highest correlation (one module for each positive and negative) was identified as the key module in DN. Accordingly, the blue module (cor = −0.74; *p* < 0.001) and yellow module (cor = 0.82; *p* < 0.001) were selected for further analysis. The blue module encompasses 2495 genes, whereas the yellow module contains 754 genes, resulting in a total of 3249 genes. The aforementioned genes were henceforth referred to as DN module genes (Figure 1F). Ultimately, the intersection of the previously obtained DEG, CSRG, and DN module genes was established, and 94 genes were found to be present in all three groups, which were subsequently identified as DE‐CSRGs (Figure 1G).

Figure 1Selection and enrichment analysis of complement system–related genes (DE‐CSRGs). (A) Volcano plot of differentially expressed genes (DEGs) in the training set. Upregulated genes are shown in orange, downregulated genes are shown in green, and nonsignificant genes are shown in gray. (B) Heatmap of DEGs in the training set. Columns represent samples, and rows represent genes; red indicates relatively high expression, and blue indicates relatively low expression. (C) Sample clustering dendrogram and trait heatmap for WGCNA. (D) Analysis of soft‐thresholding power for WGCNA. The left panel shows the scale‐free topology fit index for different soft‐thresholding powers, and the right panel shows the mean connectivity. (E) Gene dendrogram and module assignment identified by WGCNA. Different colors indicate different coexpression modules. (F) Heatmap of correlations between module eigengenes and DN status. The color scale indicates the strength and direction of the correlations, and each cell shows the correlation coefficient. (G) Venn diagram for DE‐CSRGs. (H) Gene Ontology (GO) enrichment analysis of DE‐CSRGs. Enriched GO terms are grouped into biological process (BP), cellular component (CC), and molecular function (MF) categories. The *x*‐axis shows the enriched terms, the *y*‐axis shows the number of genes enriched in each term, bar colors indicate GO categories, and bar length represents gene count. (I) Network plot of Kyoto Encyclopedia of Genes and Genomes (KEGG) enrichment analysis of DE‐CSRGs. Each node represents an enriched KEGG pathway, with node size and color intensity indicating enrichment significance. Edges between nodes represent relationships among pathways.

**Figure:**
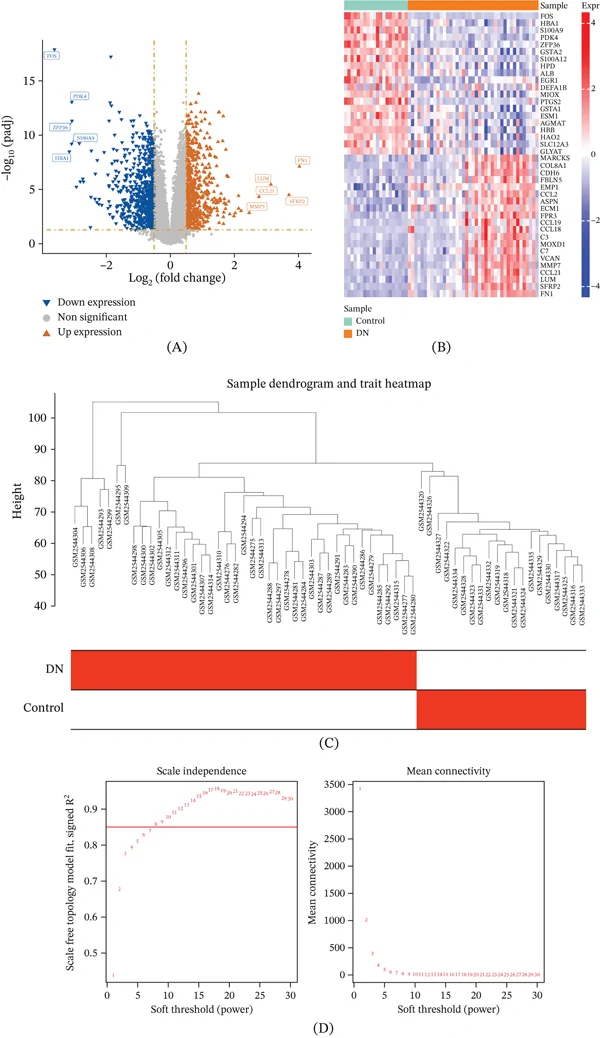

**Figure:**
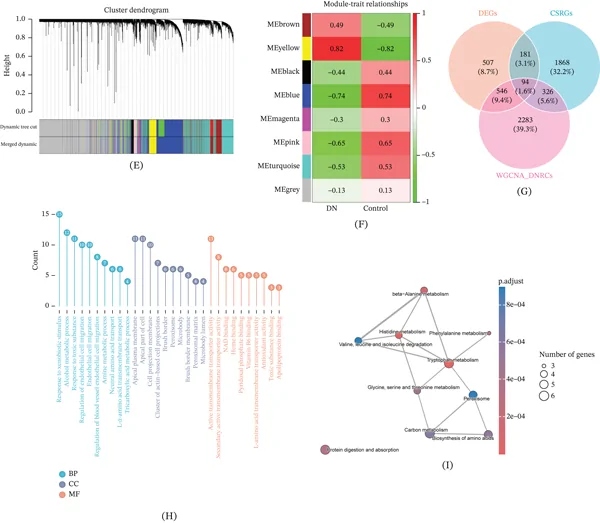

The GO analysis revealed that these genes were linked with 821 entries, in which DE‐CSRGs were associated with the biological processes of “response to external stimuli,” “apical plasma membrane,” and “DNA binding” (Figure 1H). Moreover, DE‐CSRGs were associated with 39 KEGG pathways, including tryptophan metabolism, histidine metabolism, and beta‐alanine metabolism (Figure 1I). Although canonical complement‐related pathways were not among the dominant enrichment terms, the DE‐CSRGs may still reflect complement‐associated biological alterations in DN, potentially through interactions with metabolic and inflammatory processes.

### 3.2. Acquisition of Candidate Gene for DN

**Figure 2:**
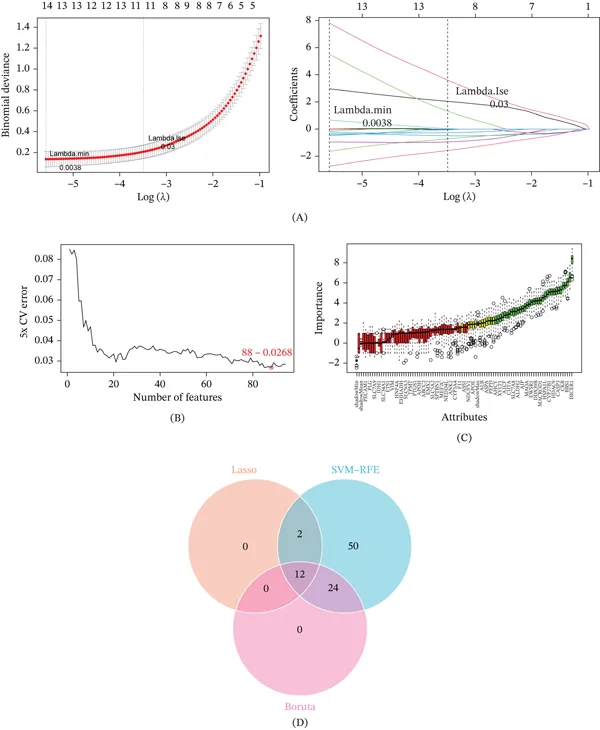
Screening of candidate genes. (A) Least absolute shrinkage and selection operator (Lasso) regression analysis. Left subplot: The *x*‐axis represents log(Lambda), a regularization parameter, and the *y*‐axis denotes cross‐validation error. As log(Lambda) increases, the cross‐validation error changes, with the red lines marking key lambda values for balancing model complexity and error. Right subplot: The *x*‐axis is log(Lambda), and the *y*‐axis shows gene coefficients. As Lambda varies, the coefficients of genes change and shrink to zero. (B) Support vector machine–recursive feature elimination (SVM‐RFE) screening candidate genes. The *x*‐axis denotes the quantity of genes, while the *y*‐axis signifies the error rate. As genes are systematically removed, the error rate varies to ascertain the ideal quantity of candidate genes that minimizes the error. (C) Boruta screens for candidate genes. Green dots represent important features (genes), yellow dots denote features with unclear importance, and the blue box shows the minimum, average, and maximum *Z*‐values of shadow attributes. (D) Venn diagram of three machine algorithm.

Machine learning approaches were employed to screen candidate genes based on DE‐CSRGs. This resulted in the identification of 14 candidate genes through Lasso regression analysis, which were subsequently classified as follows: RASA1, DICER1, TUG1, CKB, Annexin A1 (ANXA1), EWSR1, ANG, HSPA1L, CYP27B1, AIP, GTF2H5, KPNA2, OXT, and XYLT1 (Figure 2A). When the error rate of the SVM‐RFE model was 0.0268, 88 genes were identified (Figure 2B). The results of the Boruta analysis are presented in Figure 2C, and 36 genes were identified as being worthy of further investigation. After that, the genes identified were then subjected to a further round of analysis, resulting in the identification of 12 candidate genes containing RASA1, DICER1, TUG1, CKB, ANXA1, EWSR1, ANG, HSPA1L, CYP27B1, GTF2H, XYLT1, and AIP (Figure 2D).

### 3.3. Assessment of Expression and Diagnostic Capacity for Biomarkers

**Figure 3:**
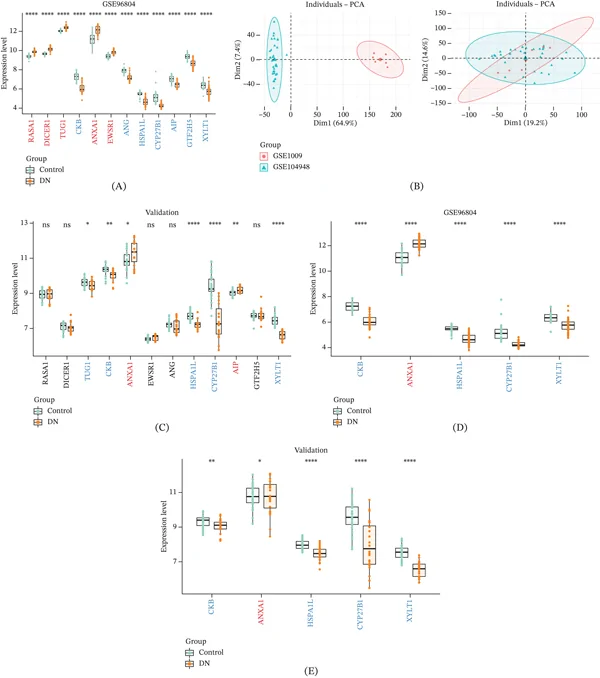
Screening and validation of biomarkers. (A) Expression boxplot of candidate genes in the training set. Genes colored red indicate upregulation in the DN group, whereas those in blue denote downregulation compared to the control group. (B) Principal component analysis (PCA) plots showing the effect of batch effect correction using ComBat. (C) Expression boxplot of candidate genes in the validation set. (D) Expression boxplot of biomarkers (exhibiting consistent expression trends as observed in both sets) in the training set. (E) Expression boxplot of biomarkers in the validation set. ns: *p* > 0.05.  ^∗^ *p* < 0.05,  ^∗∗^ *p* < 0.01, and  ^∗∗∗∗^ *p* < 0.0001.

Subsequently, a boxplot was employed to illustrate the expression disparities of the candidate genes. Compared with control groups, the expression of five genes (RASA1, DICER1, TUG1, ANXA1, and EWSR1) in the training set was markedly elevated in the DN groups, and seven genes (CKB, ANG, HSPA1L, CYP27B1, AIP, GTF2H5, and XYLT1) were significantly decreased (Figure 3A). In the combined validation set (after applying ComBat to remove batch effects, the PCA plot in Figure 3B illustrated that samples from different datasets were more evenly distributed across the plot, with reduced separation between groups, demonstrating that batch effects were effectively mitigated), compared with the control groups, the expression of ANXA1 and AIP was significantly elevated in DN groups, whereas the expression of five genes—TUG1, CKB, HSPA1L, CYP27B1, and XYLT1—was significantly diminished (Figure 3C). Based on gene expression levels, the CKB, ANXA1, HSPA1L, CYP27B1, and XYLT1 genes demonstrated consistent and significant differences in the validation set and training set. Therefore, the CKB, ANXA1, HSPA1L, CYP27B1, and XYLT1 genes were identified as biomarkers (Figure 3D,E).

### 3.4. Construction of a Nomogram by the Amount of Biomarker Expression

**Figure 4:**
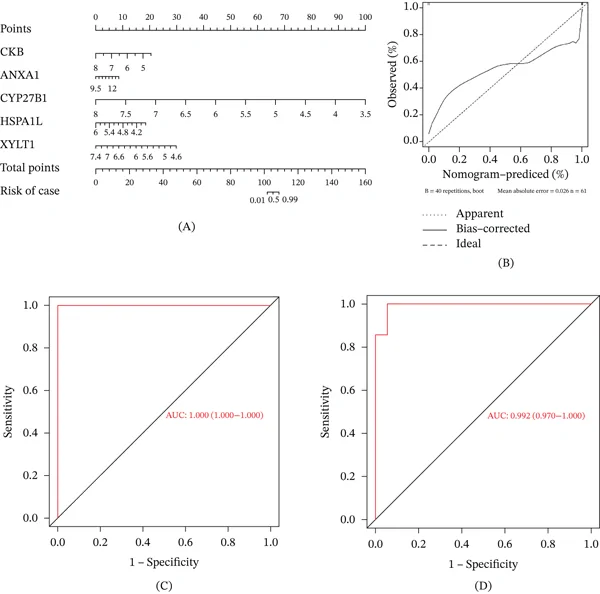
Construction of the nomogram model. (A) Construction of the nomogram model based on biomarkers. Points are assigned based on the biomarker′s expression level. The total points from all biomarkers are summed, and the corresponding “risk of case” on the bottom scale estimates the probability of being a case. (B) Calibration curve evaluation assesses the agreement between the nomogram′s predicted probabilities (*x*‐axis) and the observed actual probabilities (*y*‐axis). Closer alignment of the bias‐corrected curve to the ideal line indicates better predictive accuracy of the nomogram. (C) ROC curve to evaluate the predictive accuracy of the biomarkers in the training set GSE96804. (D) ROC curve of the nomogram model in the independent validation set GSE104948. The *x*‐axis is 1‐specificity (false positive rate), and the *y*‐axis is sensitivity (true positive rate).

In order to explore the clinical diagnostic ability of five biomarkers, we constructed a diagnostic nomogram based on the expression level of biomarkers, in which the higher the total score, the greater the probability of diagnosing disease samples (Figure 4A). Subsequently, we constructed a calibration curve and ROC curve in the training set GSE96804, the slope of which was found to be close to 1, and the AUC approached 1. The results demonstrated that the model was precise and exhibited high diagnostic efficacy (Figure 4B,C). In addition, we further validated the model in the independent validation set GSE104948, and the ROC curve showed that the AUC was 0.992, indicating that the model exhibited relatively robust predictive performance (Figure 4D).

### 3.5. GSEA‐Enriched Pathways and Gene Interactions for Biomarkers

**Figure 5:**
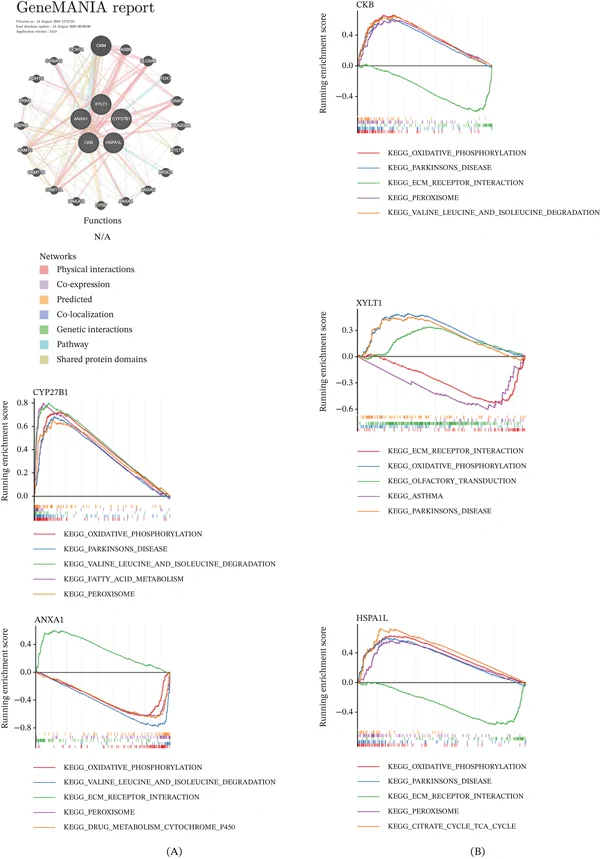
Gene Set Enrichment Analysis (GSEA)–enriched pathways and gene interactions for biomarkers. (A) Coexpression network of biomarkers by GeneMANIA. The network diagram shows the connections between the biomarkers (represented by large nodes) and other related genes (smaller nodes). (B) GSEA of biomarkers based on genes ranked by their correlation coefficients with each biomarker in the training set. The vertical bars indicate the locations of genes from each set inside the ordered list.

In order to gain further insight into the gene interaction and gene cofunction of biomarkers, a GeneMANIA network was constructed, as illustrated in Figure 5A. The network comprises five biomarkers and 20 genes that exhibit a robust interaction with the biomarkers. The relevant functions are as follows: cellular modified amino acid biosynthetic process, acid metabolism process, and lipase inhibitor activity, among others. To gain further insight into the role of different biomarkers in disease progression, we conducted a GSEA based on the training set. This revealed that the oxidative phosphorylation (OXPHOS) pathway was activated in CKB, HSPA1L, CYP27B1, and XYLT1 while it was inhibited in ANXA1 (Figure 5B and Supporting Information 2: Table S2). It was demonstrated that ATP generated by OXPHOS serves not only as an energy currency but also as an immunomodulator, initiating and amplifying the immune response [42] through paracrine and autocrine mechanisms. This finding was linked to our disease and the associated complement system.

### 3.6. Discrepancies of the Immune Microenvironment Between DN Patients and Controls

**Figure 6:**
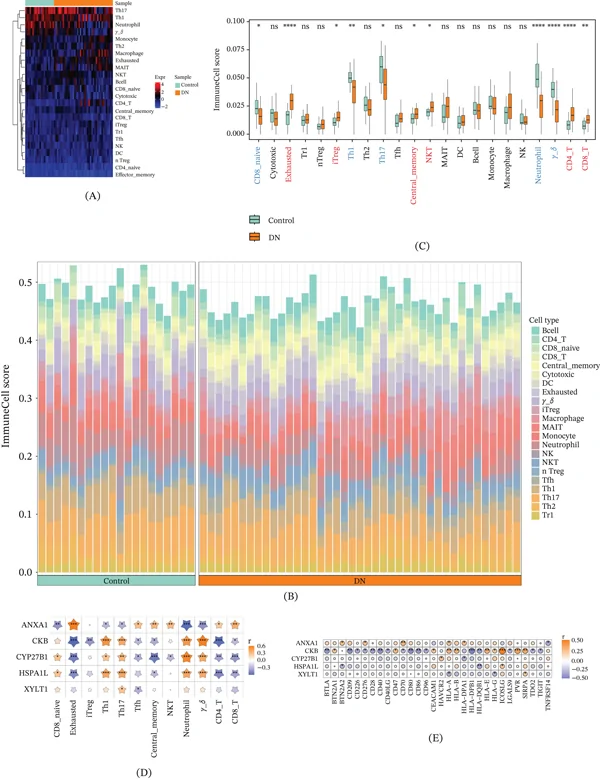
The landscape of immune infiltration between DN patients and controls. (A) Immune cell heatmap for the distribution and abundance of DN patients and controls. Immune cells that tend to have similar abundance trends across samples will cluster near each other in the clustering tree (dendrogram). (B) Histogram of immune infiltration distribution. Each column corresponds to a sample, and the color gradient represents the infiltration score of different immune cell types. It emphasizes how immune cell composition varies within individual samples. (C) Boxplot of immune infiltrating cell differences. ns: *p* > 0.05.  ^∗^ *p* < 0.05,  ^∗∗^ *p* < 0.01,  ^∗∗∗^ *p* < 0.001, and  ^∗∗∗∗^ *p* < 0.0001. (D) Heatmap displays the correlation coefficients between the infiltration levels of different immune cell types. (E) Heatmap of correlation between biomarkers and differential immune checkpoints.

In order to investigate the infiltration of immune cells in DN, the proportion of 25 immune cells was calculated, and 24 immune cells were retained after excluding immune cell types with zero infiltration in more than 50% of samples. The excluded immune cell type was effector_memory. Only eight samples showed infiltration of this cell type, whereas the remaining 53 samples had zero values, suggesting that it was not a major functional immune cell population in the specific tissue microenvironment studied here. Among them, there were 11 immune cells with significant differences after FDR correction, namely, CD8_naive, exhausted, iTreg, Th1, Th17, central_memory, NKT, neutrophil, gamma_delta, CD4_T, and CD8_T (Figure 6A,B and Supporting Information 3: Table S3). Then, we found that the strongest positive correlation was between ANXA1 and exhausted cells (cor = 0.62; *p* < 0.001), and the strongest negative correlation was between the CKB gene and the exhausted cells (cor = −0.59; *p* < 0.001) (Figure 6C,D). Immune checkpoints regulated the immune response, and a correlation analysis was conducted on the expression of 79 immune checkpoint molecules [35] in the DN sample group, as shown in Figure 6E, which illustrates the differentiated immune checkpoints. CKB demonstrated a positive and negative correlation with the majority of immune checkpoints. The most substantial positive correlation coefficient was recorded with ICOSLG (cor = 0.69; *p* < 0.001), whereas the highest negative correlation coefficient was observed with HLA‐DPB1 (cor = −0.55; *p* < 0.001) (Figure 6E). This suggests that CKB may be involved in immune regulation in DN.

### 3.7. Regulatory Networks of Biomarkers

**Figure 7:**
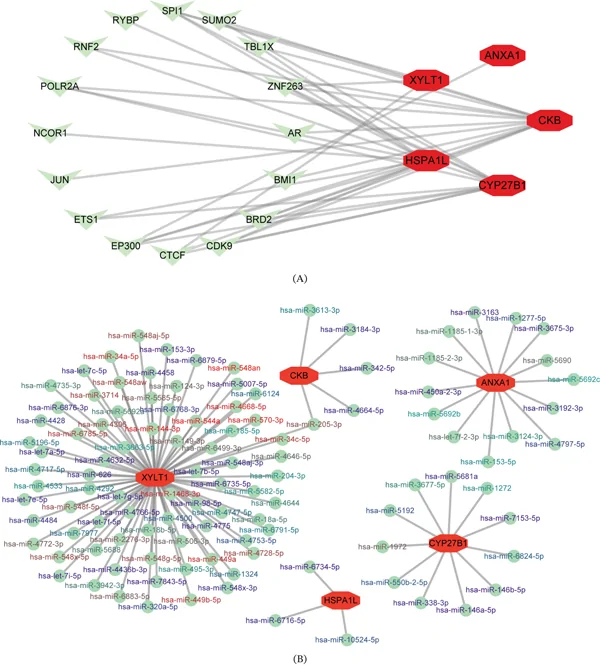
Regulatory networks of biomarkers. (A) Transcription factor (TF)–mRNA regulatory network (the red octagon was mRNA, and the green arrow shape was TF). (B) mRNA‐miRNA regulatory network (the red octagonal shapes were mRNAs, and the green circles were miRNAs; among the red labels were miRNAs with high mRNA interaction scores, and blue labels were miRNAs with low mRNA interaction scores).

To understand the interactions within the gene regulatory network, we proceeded to predict the TFs associated with the biomarkers. This resulted in the identification of a total of 16 TFs. As illustrated in Figure 7A, the network comprised a total of 21 nodes and 37 pairs of relationships, which were characterized by a high degree of interconnectedness. However, the simultaneous prediction of EP300, SPI1, and ZNF263 TFs by CKB, CYP27B1, and HSPA1L merits further investigation. Subsequently, miRNAs that were regulated by biomarkers were predicted, and the resulting network comprises 115 nodes and 111 relationship pairs (Figure 7B). Among the genes under investigation, XYLT1 demonstrated a greater number of miRNAs with higher interaction scores, indicating that this gene was subject to regulation by a greater number of miRNAs. Of particular interest was the observation that miR‐205‐3P regulated both CKB and XYLT1, a finding that merits further investigation.

### 3.8. The Relationship Between Biomarkers, Diseases, and Drugs

**Figure 8:**
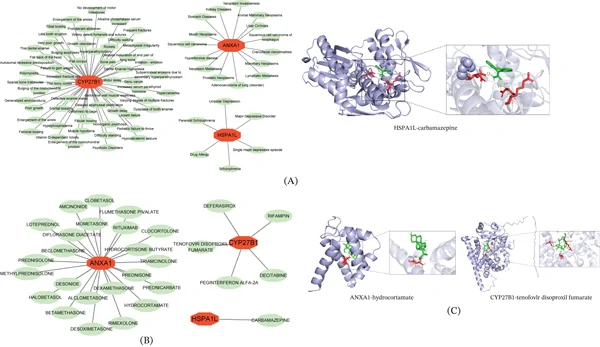
The relationship between biomarkers, diseases, and drugs. (A) Biomarker‐disease network for DN (the red octagon was mRNA, and the green arrow shape was TF). (B) Biomarker‐drug network for DN (the red octagon was mRNA, and the green arrow shape was the drug). (C) Molecular docking model diagram of HSPA1L‐carbamazepine, ANXA1‐hydrocortamate, and CYP27B1‐tenofovir disoproxil fumarate. The light blue represents the protein molecular structure, amino acids at the binding sites are shown in red, compounds are colored green, and yellow dashed lines show hydrogen bond connections.

The correlation between biomarkers and disease revealed the following: CYP27B1 was linked to an array of ailments, including polymyositis, hypercalcemia, and psychotic disorders. Nevertheless, ANXA1 was linked to a variety of tumor diseases, including squamous cell carcinoma, prostatic neoplasms, gastric diseases, and lung adenocarcinoma. HSPA1L demonstrated a stronger correlation with depression than any other factor (Figure 8A). It is noteworthy that CYP27B1 was primarily associated with immune‐related disorders, a phenomenon that bears resemblance to the DN under examination. To obtain a deeper understanding of the potential of targeted drugs for disease, a total of 29 biomarker‐related drugs were predicted. HSPA1L was associated with a single drug, namely, carbamazepine. CYP27B1 was linked to five drugs: decitabine, deferasirox, rifampin, tenofovir disoproxil fumarate, and peginterferon alfa‐2a. ANXA1 was associated with a greater number of drugs, including hydrocortamate, desoximetasone, and rimetolone (Figure 8B). Subsequently, molecular docking was conducted on the drugs with the highest predicted scores for the aforementioned three biomarkers. The docking results suggested possible binding interactions between the selected compounds and the three biomarkers (Table 1). The results demonstrated that the binding energy of HSPA1L and carbamazepine was −8.9, that of ANXA1 and hydrocortamate was −6.6, and that of CYP27B1 and tenofovir disoproxil fumarate was −6.8 (Figure 8C).

**Table 1:** Molecular docking binding energy of the core target.

Symbol	PDB/UniProt	Molecule name	PubChem CID	Affinity (kcal/mol)	Hydrogen bonds
HSPA1L	3GDQ	Carbamazepine	2554	−8.9	3
ANXA1	1QLS	Hydrocortamate	84088	−6.6	1
CYP27B1	O15528	Tenofovir disoproxil fumarate	6398764	−6.8	3

### 3.9. HSPA1L Identified as a Risk Factor for DN

**Figure 9:**
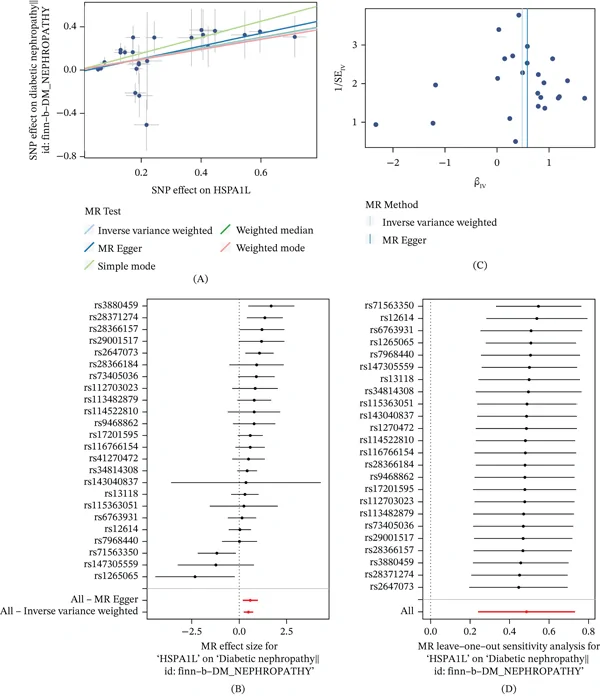
HSPA1L identified as a risk factor for DN. (A) Scatter plot of the causality of HSPA1L and DN. Each point represents a single SNP, with the *x*‐axis showing the SNP effect on HSPA1L and the *y*‐axis representing the SNP effect on DN. Different lines depict various MR methods used to estimate the causal effect. (B) Forest diagram of the causal effects (via the Wald ratio method) of HSPA1L and DN. Each horizontal solid line corresponds to a single SNP′s estimated result: If a line lies entirely to the left of 0, it indicates that an increase in the exposure factor (HSPA1L) reduces the risk of the outcome (DN); if a line lies entirely to the right of 0, an increase in HSPA1L elevates the risk of DN. The red horizontal lines represent the summary causal effect estimates of all included SNPs. (C) Funnel plot of HSPA1L and DN supports the robustness of the MR results (no major bias/heterogeneity). It uses two MR methods (IVW and MR‐Egger), with the *x*‐axis representing the effect size and the *y*‐axis showing 1/SE (inverse standard error). (D) Leave‐one‐out plot of HSPA1L and DN. Each row corresponds to the model′s effect when a specific SNP is removed. The red horizontal lines denote the overall causal effect estimate when all SNPs are included.

To further elucidate the causal relationship between biomarkers and DN, CKB, ANXA1, HSPA1L, CYP27B1, and XYLT1 were analyzed as exposure traits, with DN serving as the outcome in an MR analysis. Subsequently, a total of 101 SNPs for the five biomarkers were identified (Supporting Information 4: Table S4). After clumping, harmonization, removal of weak instruments, and excluding biomarkers with fewer than three valid IVs, XYLT1, ANXA1, CKB, and HSPA1L were retained for MR analysis. The final number of SNPs included for each exposure is presented in Supporting Information 4: Table S4. The IVW method demonstrated a significant causal relationship between HSPA1L and DN and established that HSPA1L was a risk factor for DN (OR = 1.625, 95% CI: 1.272–2.076, *p* = 9.96e − 05) (Table 2). The scatter plot demonstrated a positive correlation between HSPA1L expression and the elevated risk of DN (Figure 9A), whereas the forest plot illustrated that the effect size of HSPA1L on DN was consistently greater than zero (Figure 9B). The funnel plot showed that the SNP‐specific causal estimates were approximately symmetrically distributed around the overall effect estimate, with no obvious asymmetry (Figure 9C), suggesting no strong evidence of directional horizontal pleiotropy. Cochran′s *Q* test indicated heterogeneity (*p* < 0.05) (Table 3), suggesting possible between‐instrument heterogeneity in the causal estimates. Therefore, the random‐effects IVW model was applied. In addition, the pleiotropy test showed no significant evidence of directional horizontal pleiotropy (*p* > 0.05) (Table 4). The LOO analysis demonstrated that the impact on DN remained relatively consistent after the exclusion of each SNP (Figure 9D). Finally, a directivity test was conducted, which yielded a positive result, as evidenced in Table 5. The HSPA1L gene demonstrated the capacity to pass the directivity test without exhibiting reverse influence. After correction for multiple testing, HSPA1L remained significantly associated with DN (*p* < 0.05) (Table 6), whereas the other exposure traits were not significant, further supporting the robustness of the inferred causal association.

**Table 2:** Mendelian randomization (MR) analysis of HSPA1L and DN.

Outcome	Exposure	Method	NSNP	*b*	SE	*p*val	OR	or_lci95	or_uci95
Diabetic nephropathy || id:finn‐b‐DM_NEPHROPATHY	ENSG00000204390 || id:eqtl‐a‐ENSG0000024390	MR‐Egger	24	0.584	0.201	0.008	1.794	1.211	2.658
		Weighted median	24	0.490	0.137	0.000	1.633	1.247	2.137
		Inverse variance weighted (multiplicative random effects)	24	0.486	0.125	0.000	1.625	1.273	2.076
		Simple mode	24	0.738	0.238	0.005	2.091	1.312	3.332
		Weighted mode	24	0.461	0.169	0.012	1.585	1.137	2.210

*Note:* *p*val, significance.

Abbreviations: *b*, beta; CI, confidence interval; heter_P, heterogeneity significance; NSNP, number of single‐nucleotide polymorphisms used for analysis; OR, odds ratio; pleio_P, horizontal pleiotropy significance; SE, standard error.

**Table 3:** The results of the heterogeneity test between HSPA1L and DN.

Outcome	Exposure	Method	*Q*	*Q*_df	*Q*_*p*val
Diabetic nephropathy || id:finn‐b‐DM_NEPHROPATHY	ENSG00000204390 || id:eqtl‐a‐ENSG00000204390	MR‐Egger	38.797	22	0.015
		Inverse variance weighted	39.506	23	0.021

*Note:* Method: analysis test method (focus on IVW method). *Q*, Cochran′s *Q* heterogeneity statistics; *Q*_df, degrees of freedom; *Q*_*p*val, significance.

**Table 4:** The results of the horizontal pleiotropy test between HSPA1L and DN.

Outcome	Exposure	Egger_intercept	SE	*p*val
Diabetic nephropathy || id:finn ‐b‐DM_NEPHROPATHY	ENSG00000204390 || id:eqtl‐a‐EN SG00000204390	−0.019	0.030	0.533

*Note:* Egger_intercept: indicates the intercept value. *p*val: significance.

Abbreviation: SE, standard error of intercept.

**Table 5:** Directionality test.

Exposure	Outcome	snp_r2.exposure	snp_r2.outcome	Correct_causal_ direction	Steiger_*p*val
ENSG00000204390 || id:eq tl‐a‐ENSG00000204390	Diabetic nephropathy || id:finn‐b‐DM_NEPHROPATHY	0.241	0.0003	True	0

**Table 6:** Multiple‐testing correction of MR results for candidate biomarkers.

Gene	*p* value	*p*.adj
XYLT1	0.400073988008971	0.533431984011961
ANXA1	0.0924645646436826	0.184929129287365
CKB	0.938219071469907	0.938219071469907
HSPA1L	9.96354917206384e‐05	0.000398541966882554

## 4. Discussion

The complement system has been associated with the pathophysiology of DN, a primary contributor to end‐stage renal disease globally. A comprehensive genomic profiling study has shown that the renal C3 complement component may promote the progression of DN [43]. This viewpoint is supported by another study [44], which identified key genes related to the complement cascade, such as C1QA, C1QB, C3, CFB, ITGB2, VSIG4, and CLU, that may play significant roles in the occurrence and development of DN. Furthermore, research indicates a mechanistic link between intrarenal SGLT‐2 and complement C5 synthesis in DN [45]. Another study investigated complement deposition in the renal tissue of patients with DN, further supporting the involvement of the complement system in this disease [46]. The literature suggests that the complement system may be integral to the pathophysiology of DN. Acquiring a more profound comprehension of the mechanisms governing complement activation in diabetes may provide significant insights for prospective treatment strategies. In the context of DN, complement‐associated genes may contribute to disease progression through cross‐talk with metabolic and inflammatory pathways [47], which may partly explain why metabolism‐related terms were prominently enriched.

In our study, we identified complement system–related biomarkers for DN through bioinformatics analysis, including CKB, ANXA1, HSPA1L, CYP27B1, and XYLT1. The ANXA1 gene plays a crucial role in inflammation, immune responses, and cancer biology [48]. ANXA1 promotes the resolution of inflammation by inhibiting the recruitment of neutrophils and enhancing their apoptosis, thereby preventing excessive inflammatory responses that can lead to tissue damage [49, 50]. This is particularly important in DN, a common complication. Research has shown that patients with DN have elevated levels of ANXA1 in their kidneys, and this elevation is associated with renal function and prognosis. Further studies using mouse models indicate that the absence of ANXA1 exacerbates kidney damage in diabetic mice, whereas overexpression of ANXA1 can alleviate this damage, suggesting that ANXA1 may serve as a potential therapeutic target for DN [49]. Additionally, another study highlights the potential of ANXA1 to regulate lipid metabolism through the AMPK/PPAR*α*/CPT1b pathway. The research indicates that the loss of ANXA1 worsens kidney injury and promotes lipid accumulation within the kidneys, whereas ANXA1 mimetic peptides show therapeutic effects against lipid toxicity in diabetic mice [51]. This suggests that ANXA1 not only contributes to the resolution of inflammation but also plays a role in metabolic regulation, providing further insights into its potential as a therapeutic target.

The HSPA1L gene, part of the heat shock protein (HSP) family, has been implicated in various cellular processes, including protein folding, protection against stress, and immune response regulation [52]. The Vaspin‐HSPA1L complex can protect proximal tubular cells from organelle stress in DN [53]. However, there is relatively little literature currently linking HSP to DN. A study conducted on a South Indian population identified specific single‐nucleotide polymorphisms in the HSP70 family (including HSPA1L) associated with renal complications of T2D [53]. This study emphasized the correlation between the HSP70 family, including HSPA1L, and renal function in patients with DN. In our research, the MR results indicated that HSPA1L is a risk factor for DN (OR = 1.625, 95% CI: 1.272–2.076, *p* = 9.96e − 05), highlighting its correlation with DN.

CYP27B1 is an enzyme that catalyzes the conversion of 25‐hydroxyvitamin D into its active form, 1,25‐dihydroxyvitamin D (calcitriol) [54]. Research has found that allele B (BB or Bb genotype) of the vitamin D receptor gene correlates with substantial proteinuria in the Han Chinese demographic with T2D and may serve as a risk factor for early‐onset DN [55]. Additionally, studies have shown that the CC genotype of the CYP27B1 polymorphism increases the risk of Type 1 diabetes, suggesting a potential link between genetic variations in CYP27B1 and diabetes‐related complications [56]. There is evidence that vitamin D status, influenced by CYP27B1 activity, may be related to complement component levels through the TLR2‐dependent p38/MAPK‐CYP27B1‐VDR‐CAMP pathway [54, 57]. Therefore, we hypothesize that the CYP27B1 gene may affect vitamin D metabolism, thereby influencing immune responses and modulating the role of the complement system in DN. Unfortunately, there are currently no reports linking CKB and XYLT1 to DN. However, both are engaged in the control of the complement system. For instance, CKB, also known as brain‐type creatine kinase (CK‐BB), is a crucial enzyme involved in cellular energy metabolism that phosphorylates glutathione peroxidase 4 (GPX4) S104 [58]. Studies have shown that CKB impedes epithelial–mesenchymal transition (EMT) and the advancement of prostate cancer via inhibiting AKT activation, which is a key pathway in inflammation and immune‐related cellular processes [59]. This suggests that CKB may have a broader role in regulating inflammatory pathways and immune responses. Furthermore, the epigenetic regulation of CKB is associated with obesity‐related inflammation. In white adipose tissue, endoplasmic reticulum stress has been shown to inhibit the transcription of CKB, leading to an increase in proinflammatory factors such as CCL2 [60]. This highlights CKB as a potential therapeutic target for treating inflammation associated with metabolic disorders. XYLT1 is an enzyme that catalyzes the transfer of xylose to serine residues on the core protein, a key step in the synthesis of glycosaminoglycans and proteoglycans [61]. Research indicates that XYLT1 is involved in the retention of mutant proteins in the endoplasmic reticulum, and this retention may trigger stress responses and further activate inflammatory pathways [62]. We hypothesize that CKB and XYLT1 may be involved in the inflammatory and immune dysregulation associated with the complement system. Subsequent studies will experimentally validate their specific roles in DN.

GSVA demonstrated that the OXPHOS pathway is closely related to these biomarkers. The OXPHOS pathway drives the production of mitochondrial ATP, and mitochondria, as dynamic organelles, maintain renal homeostasis in diabetes through continuous fusion and fission [63]. In the drug prediction analysis, CYP27B1 was computationally associated with rifampin. Studies have shown that rifampicin and its analogs can reduce the formation of advanced glycation end‐products, potentially alleviating some harmful effects of diabetes. In a mouse model of obesity‐induced T2D, it was found that rifampicin could lower HbA1c levels and improve insulin sensitivity, indicating its protective role in preventing the occurrence of DN without causing hepatotoxicity [64]. However, these drug–target associations and docking results are based solely on bioinformatics prediction and in silico binding simulations and therefore do not demonstrate disease‐specific efficacy or therapeutic potential in DN without further experimental validation.

Immune infiltration studies revealed differences in various immune cells associated with DN, such as CD8+ T cells and Th1 cells. CD8+ T cells are cytotoxic T cells that can directly kill target cells and regulate immune responses by secreting cytokines. Research has demonstrated that mesenchymal stem cells (MSCs) can mitigate DN by inhibiting CD8+ T cell responses. In DN models, MSC transplantation has been shown to reduce renal CD8+ T cell infiltration, thereby diminishing renal injury and fibrosis [65]. Notably, in the context of age‐related macular degeneration, ANXA1 was found to be elevated and associated with CD8+ T cell immune cells [66], suggesting a role in regulating the immune response. Additionally, Th1 cells promote inflammatory responses by secreting proinflammatory cytokines, including IFN‐*γ* and TNF‐*α*, hence expediting the evolution of DN [67, 68]. The study uncovers the connection between immune response and the progression of DN, offering fresh insights into the inflammatory mechanisms underlying this condition.

However, this study has several limitations. First, our research primarily relies on public gene databases, which may not offer sufficient depth of clinical/molecular information and lack experimental validation. Moreover, the CSRGs analyzed in this study were obtained from the GeneCards database. However, the GeneCards‐derived definition may be broader than strictly curated canonical complement gene sets, as it may include genes with indirect associations. Therefore, the biological specificity of the selected complement‐related genes should be interpreted with caution. Second, the sample sizes of individual (and even integrated) public datasets are relatively small—this may introduce biases in statistical inference and limit the generalizability of our findings. Therefore, future work will use expanded clinical specimens (with larger sample sizes and comprehensive metadata) and DN animal models to further elucidate the specific roles of different components of the complement system in the progression of DN. Finally, with regard to MR analysis, we acknowledge a potential limitation related to SNP selection: The current IV selection criteria could be further optimized by applying stricter thresholds (e.g., *p* < 5 × 10^−8^) in future studies. In addition, although multiple‐testing correction was performed and only HSPA1L remained significant after adjustment, the number of exposure traits included in the MR analysis was limited, and the IVs were selected using a relatively lenient threshold. Therefore, the MR findings should still be interpreted with caution and further validated in larger clinical cohorts and more diverse populations.

## 5. Conclusions

In summary, through integrated bioinformatics analysis of three public datasets (GSE96804, GSE104948, and GSE1009) and validation via multiple approaches, we found that complement system–related CKB, ANXA1, HSPA1L, CYP27B1, and XYLT1 may significantly influence the advancement of DN. Among these, MR further confirmed HSPA1L as a causal risk factor for DN, while the diagnostic nomogram constructed based on the five CSRGs exhibited high accuracy, supporting their potential as clinical diagnostic biomarkers for DN. Collectively, the findings of this study establish a foundation for additional investigation into the pathophysiology of DN (especially a new direction for studying the role of the complement system in its progression) and provide potential molecular markers for its clinical diagnosis. Additionally, these findings may provide a preliminary basis for future studies exploring drug–target interactions related to complement‐associated pathways in DN. However, their therapeutic relevance requires further experimental and clinical validation.

**Nomenclature**
CKD: chronic kidney disease
CSRGs: complement system–related genes
DE‐CSRGs: differentially expressed CSRGs
DEGs: differentially expressed genes
DN: diabetic nephropathy
EMT: epithelial–mesenchymal transition
ESKD: end‐stage kidney disease
FDR: false discovery rate
GO: Gene Ontology
GSVA: gene set variation analysis
KEGG: Kyoto Encyclopedia of Genes and Genomes
Lasso: least absolute shrinkage and selection operator
MR: Mendelian randomization
MSCs: mesenchymal stem cells
OXPHOS: oxidative phosphorylation
ROC: receiver operating characteristic
SVM‐RFE: support vector machine–recursive feature elimination
T2D: Type 2 diabetes

## Author Contributions

Study conceptualization: Lifang Wei and Yuhui Lu. Data analyses: Ye Li. Data interpretation: Yue Yu and Minmin Xu. Supervision: Jing Cai. Writing—original draft: Lifang Wei. Writing—review and editing: all authors.

## Funding

This study was funded by the Natural Science Foundation of Fujian Province (10.13039/501100003392) (2023J01885), the key subject of Fujian University of Traditional Chinese Medicine (X2023029), and the Fujian University of Traditional Chinese Medicine 2023 High‐Level Key Discipline of Traditional Chinese Medicine (Health Management in Traditional Chinese Medicine), and Provincial‐Level Key Clinical Specialty Construction Project (Geriatrics Department) (XJG2023020), Hospital‐Level Key Discipline Project of the Third Peoples′ Hospital Affiliated to Fujian University of Traditional Chinese Medicine (2025ZD002).

## Ethics Statement

The authors have nothing to report.

## Conflicts of Interest

The authors declare no conflicts of interest.

## Supporting Information

Additional supporting information can be found online in the Supporting Information section.

## Supporting information


**Supporting Information 1** Table S1: Summary of datasets and analytical roles included in this study.

**Supporting Information 2** Table S2: Gene Set Enrichment Analysis (GSEA)–enriched pathways for biomarkers.

**Supporting Information 3** Table S3: Differential analysis of immune cell infiltration between DN and control groups.

**Supporting Information 4** Table S4: Screening results of SNP by Mendelian randomization analysis.

## Data Availability

The datasets generated and/or analyzed during the current study are available in the GEO dataset (Accession Numbers: GSE96804 and GSE104948, https://www.ncbi.nlm.nih.gov/gds) and the IEU OpenGWAS database (https://gwas.mrcieu.ac.uk/).
