# Supplementary material for: Complement System–Related Genes in Diabetic Nephropathy: Screening for Potential Targets of the Mechanism
Source: J Diabetes Res. 2026 Jul 5;2026:7117751. doi: 10.1155/jdr/7117751 (PMC13599590; doi:10.1155/jdr/7117751)
Supplement: Supplementary file 1 [file JDR-2026-7117751-s003.docx]

Table S1 Summary of datasets and analytical roles included in this study

| **Dataset / Resource** | **Platform** | **Sample type** | **DN (n)** | **Control (n)** | **Role in analysis** |
| --- | --- | --- | --- | --- | --- |
| GSE96804 | GPL17586 | Glomerular tissue | 41 | 20 | Training set |
| GSE104948 (GPL22945) | GPL22945 | Renal tissue | 7 | 18 | Validation cohort |
| GSE104948 (GPL24120) | GPL24120 | Renal tissue | 5 | 3 | Validation cohort |
| GSE1009 | GPL8300 | Renal tissue | 3 | 3 | Validation cohort |
| Integrated validation set | ComBat-corrected merged dataset | Renal tissue | 15 | 24 | External validation |
| GeneCards | v5.20 | Gene database | — | — | Source of complement system-related genes |
| finn-b-DM_NEPHROPATHY | IEU OpenGWAS | European population | 3,283 | 210,163 | MR analysis |
